# Infection prevention and ultrasound probe decontamination practices in Europe: a survey of the European Society of Radiology

**DOI:** 10.1007/s13244-016-0528-z

**Published:** 2016-10-24

**Authors:** Christiane Marita Nyhsen, Hilary Humphreys, Carlos Nicolau, Gerhard Mostbeck, Michel Claudon

**Affiliations:** 1 Radiology Department, City Hospitals Sunderland, Kayll Road, Sunderland, SR4 7TP UK; 2 Royal College of Surgeons in Ireland, RCSI Education & Research Centre, Beaumont Hospital, Beaumont Dublin 9, Ireland; 3 Hospital Clinic de Barcelona, Villarroel 170, ES 8036 Barcelona, Spain; 4 Wilhelminenspital, Montleartstr. 37, AT 1160 Vienna, Austria; 5 Radiology Department, Hôpital de Brabois, CHRU de Nancy and IADI INSERM U 947, Rue du Morvan, FR 54511 Vandoeuvre Les Nancy Cedex, France

**Keywords:** Ultrasound, Gels, Infection prevention and control, Decontamination, Disinfection

## Abstract

**Objectives:**

Although ultrasound (US) is considered one of the safest imaging modalities, concerns have been raised regarding potential infection transmission risks through US procedures. A survey was undertaken by the European Society of Radiology (ESR) to establish infection prevention and control measures in US and to highlight the importance of good medical practice.

**Methods:**

An online survey was sent to all 22,000 full ESR members.

**Results:**

The response rate of completed surveys was 4.3 % (946 practitioners, 97 % of which were radiologists, mostly working in larger hospital settings). Among respondents, 29 %, 11 % and 6 % did not disinfect the US probe after every patient when performing standard surface US, endo-cavity US and interventional procedures, respectively. Eleven percent did not always use probe covers for endo-cavity US; for interventional procedures, the proportion was 23 %. A minority used sterile gel sachets in direct patient contact for endo-cavity scans (30 %), and 77.5 % used sterile gel for interventional procedures.

**Conclusions:**

The survey results highlight a wide range of practices throughout Europe and the need to raise awareness amongst practitioners regarding the importance of infection prevention and control measures. The development of European recommendations encompassing all US examinations, together with education is a priority.

***Main Messages*:**

• *Transmission of infection through ultrasound procedures is possible*.

• *There is a wide range of ultrasound probe decontamination practices in Europe*.

• *Not all practitioners use probe covers for endo-cavity or interventional ultrasound*.

• *Not all practitioners use sterile gel for internal and invasive procedures*.

• *Currently there are no European recommendations encompassing all US examinations*.

**Electronic supplementary material:**

The online version of this article (doi:10.1007/s13244-016-0528-z) contains supplementary material, which is available to authorized users.

## Introduction

Ultrasound (US) is generally considered one of the safest diagnostic modalities available. Neither professionals nor patients undergoing US imaging anticipate that US will cause significant harm. However, in recent years, several outbreaks of infection related to endoscopic procedures or trans-oesophageal US have been reported [1–5], resulting in the introduction of more stringent infection prevention measures [6, 7].

Concerns have been raised that transmission of infection may also pose a risk to patients in conventional US, in particular endo-cavity (i.e., trans-vaginal or trans-rectal) US, performing US near wounds/stomas, or interventional procedures involving US. Furthermore, there is a potential risk of transmitting infection through US gel, and the risk of microscopic or macroscopic probe cover perforations necessitates consideration.

It is very difficult to prove contamination and the subsequent transmission of pathogens through US examinations, but it should not be assumed that the risk does not exist. Surveys in the UK have already revealed that there is a wide range of practices, with the authors calling for national guidance [8, 9]. To our knowledge, only European guidelines relating to interventional US have been published [10]; no European guidance is yet available encompassing all US settings.

We report the results of a survey undertaken by the European Society of Radiology (ESR) to identify current European US infection prevention and control practices in place and to raise awareness of the importance of appropriate decontamination protocols.

## Materials and methods

An online survey (using the SurveyMonkey software) was sent to all full ESR members in Europe (22,000) in September 2015.

A total of 22 questions were posed, initially regarding the country and city of work, the work setting (private centre versus hospital), size of the establishment and number of yearly US procedures undertaken. Thereafter, known incidents involving transmission of infection were queried, with free text available for further clarification.

The final part of the survey gathered detailed data on the US gel used, both in direct contact with the patient and inside probe covers. Decontamination procedures were surveyed over a wide range of US examinations: standard surface US, when scanning patients with known transmissible infections, endo-cavity (trans-vaginal and trans-rectal) US, and US used in an interventional setting (US-guided biopsy, drainage or US used in theatre). Free text sections were available throughout the survey (see Appendix 1).

## Results

Of the 22,000 questionnaires sent, a total of 1088 replies (4.94 %) were received, of which 946 (4.3 %) were complete and subsequently analysed. Responses were gathered from most European countries, with the largest numbers originating from Italy, the UK, Spain and France.

The majority of survey respondents worked exclusively in a hospital setting (81 %); the remainder worked in private centres or offices (15 %), and 4 % indicated mixed/other work commitments. Most practitioners (67 %) worked in relatively large institutions (100–1000 beds), with 16 % working in very large hospitals of over 1000 beds. Twelve percent had no patient admission facilities. The majority (88 %) performed over 3000 US examinations per year, with 42 % of the institutions carrying out over 10,000 annual scans.

Almost all surveys were completed by radiologists (97 %). Eight responses in total were received from cardiologists, gynaecologists/obstetricians and urologists. Twenty replies stated mixed practice or work in other specialties such as breast services, paediatrics or emergency medicine.

### Known cases of infection transmission through ultrasound

Of the 946 respondents, 26 (2.7 %) stated that they were aware of cases of infection transmission through US procedures. Details provided in the free text section mentioned insufficient infection prevention measures several times. Presumed transmitted organisms included methicillin-resistant *Staphylococcus aureus* (MRSA), *Staphylococcus*, cytomegalovirus and human papillomavirus, as well as cutaneous fungal infection and scabies. Infections were recorded after breast biopsy/drainage, following joint aspiration/injection, following trans-vaginal US and after trans-rectal biopsy (the latter of which is well recognised given the presence of bowel flora). A few cases described skin infections and complications when assessing wounds as well as gastroenteritis/gastroenterocolitis. Others mentioned MRSA without offering further details.

Reviewing this subgroup in comparison to overall results did not reveal relevant differences in infection prevention measures recorded.

### Surface ultrasound on unbroken skin (i.e., presumed relatively low-risk procedures)

Most practitioners used either refill US gel bottles (520, 55 %) or single-use bottles (405, 43 %). Only 2 % used sterile sachets.

**Fig. 1:**
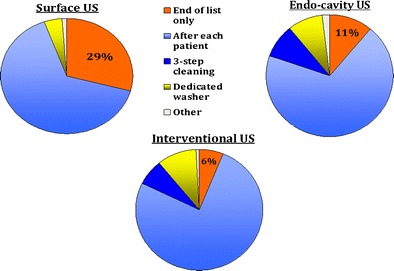
US probe decontamination

Almost two thirds of respondents decontaminated the US probe by wiping off the US gel first with subsequent disinfection of probes with foam or wipe after each patient (618, 65 %), whilst 276 (29 %) disinfected the probes only at the end of the list. Only 4 % used a dedicated washer to thoroughly decontaminate probes in this setting (see Fig. 1). A few responses detailed other protocols or gave insufficient information.

In the free text section, some practitioners stated that they wiped the probe with a dry towel but that no subsequent disinfection measures were undertaken. A multitude of different wipes and foam brands were detailed, and two respondents mentioned the use of ultraviolet light sterilisation, but this question was not answered by 803 respondents.

### Cases with known infections (surface ultrasound on unbroken skin)

When the patient was known to have a transmissible infection (infection control cases), 724 (77 %) wiped off the US gel and then used disinfecting wipes/foam after the procedure, and 174 (18 %) cleaned the probe in a dedicated washer. Forty-eight (5 %) detailed other procedures or gave insufficient information.

In the free text section, 60 respondents stated that in these cases, they would use probe covers as an additional preventative step, but not all disinfected the probe after removal of the protective sheath.

### Endo-cavity ultrasound procedures (trans-vaginal or trans-rectal ultrasound)

**Fig. 2:**
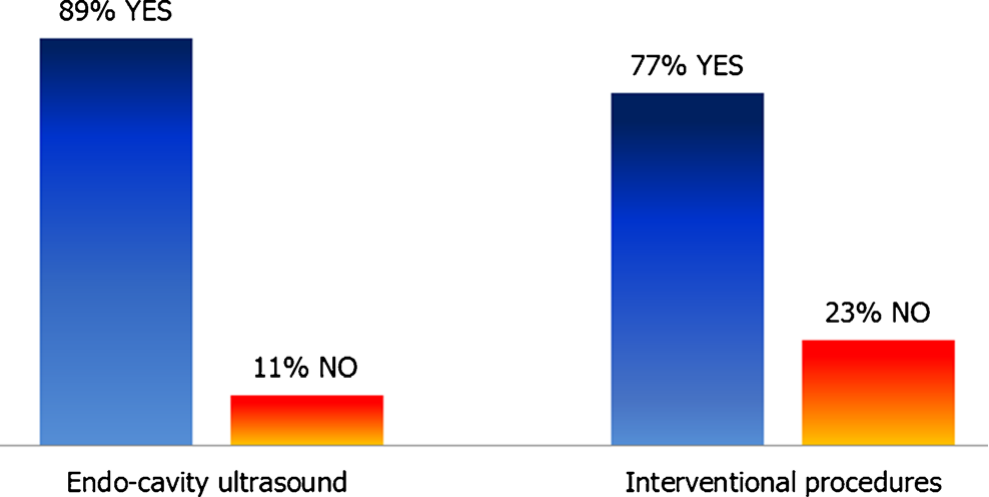
Use of US probe covers at all times

Most practitioners stated that they used a probe cover for endo-cavity US scans at all times (697, 89 %), whilst 85 (11 %) did not (see Fig. 2). Unfortunately, 164 respondents did not answer this question, possibly because they did not perform these procedures.

**Fig. 3:**
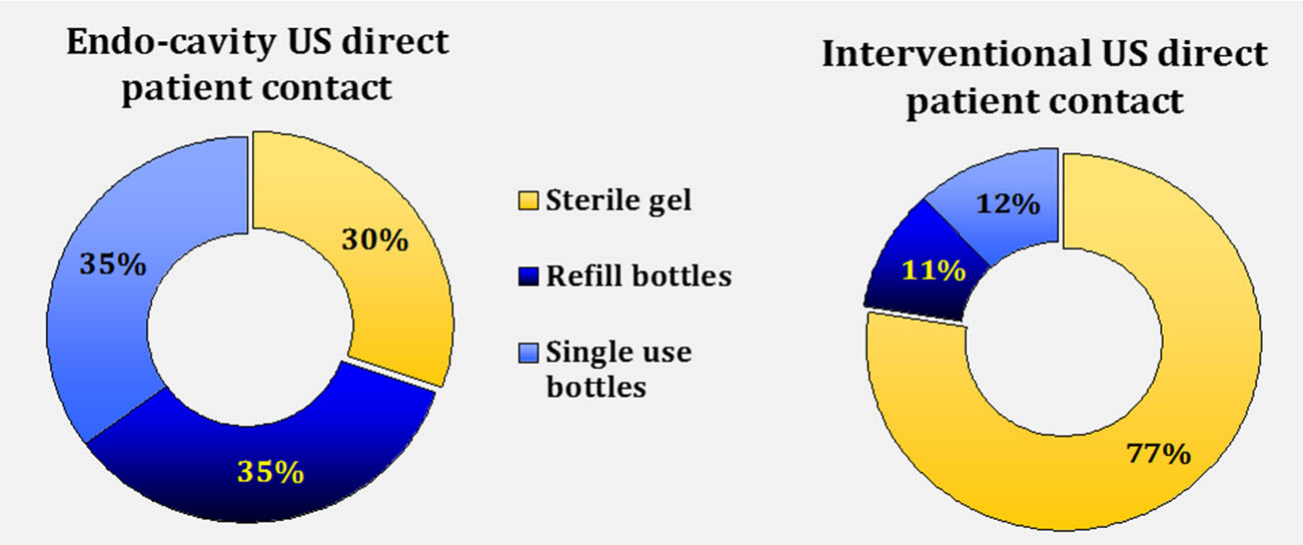
US gel used

A minority (110 respondents, 14 %) used sterile gel inside the probe cover; most used either refill bottles (355, 46 %) or single-use bottles (304, 40 %). A total of 177 respondents skipped this question. Nearly a third, 233 (30 %), stated that they used sterile gel in direct patient contact for endo-cavity procedures (233, 30 %). Refill bottles were used by 269 (35 %), 271 (35 %) used gel from single-use bottles (see Fig. 3), and 173 skipped this question.

With regard to endo-cavity US probe decontamination procedures, the majority of respondents (69 %) chose “wiping off ultrasound gel and disinfection after each patient with foam/wipe”. A more thorough “3-step cleaning process” with high-level disinfectant wipes or “probe cleaning in dedicated washer” was chosen by 69 and 70 (9 %) practitioners, respectively. Just over one in ten (86, 11 %) cleaned the probe only after the list was finished and not after each patient (see Fig. 1). A few practitioners detailed other procedures, and 166 skipped this question.

### Interventional procedures

Over two thirds of practitioners taking part in this survey stated that they performed interventional procedures (70 %). Of these 666 interventional practitioners, the majority used probe covers at all times (513, 77 %), whilst almost one quarter (153, 23 %) did not (see Fig. 2).

The US gel inside the probe cover used for interventional procedures was typically from either a refill bottle (269, 40.4 %) or a single-use bottle (229, 24.4 %), whilst 168 (25.2 %) used sterile sachets. US gel used in direct contact during interventional procedures was mostly from sterile sachets (516, 77.5 %). The remainder stated they used gel from refill bottles (70, 10.5 %) or single-use bottles (80, 12 %) as show in Fig. 3.

US probe decontamination procedures were similar to endo-cavity probe disinfection, with most practitioners wiping off the gel with subsequent disinfection after each patient (507, 76 %), whilst 47 (7 %) used a thorough three-step high-level disinfectant wipe cleaning process, and 66 (10 %) used a dedicated probe washer (see Fig. 1). A minority did not disinfect the probe after each patient, but only after the list was finished (40, 6 %). Again, some practitioners detailed other procedures which did not fit into the categories above or provided insufficient information. Several responses stated that probe covers were used but that the probes were not disinfected afterwards.

## Discussion

Publications dating back to 1988 have raised concerns regarding cross-infection risks of US procedures [11]. A case report from the year 2000 describes the transmission of hepatitis C during US-guided procedures in the setting of assisted conception, the exact route of transmission being unclear [12]. Several other studies have highlighted that post-procedure US probe contamination is significant, and that transmission of infection is possible [8, 13–18]. Reviewing the literature concerning US-guided procedures, an overall incidence of infectious complications of 0.1 % has been reported [19]. This rate may increase to 0.19 % after trans-bronchial endoscopic US-guided needle aspiration [20] and to 4.29 % after trans-rectal prostate biopsy [21].

Infections may be clinically silent and discovered only at a later date, potentially not connected to previous US examinations. Obtaining proof that an infection is due to previous US investigations can be difficult unless incidents emerge with a traceable originating source. Also, some incidents of infection transmission will not have been published.

The survey reported here confirms a very wide range of practices throughout Europe. Twenty-six practitioners stated that they knew of cases of infection transmission through US procedures, proving that this is a topic worth considering. The actual figure of US probe contamination and transmission of infection is unknown, and is potentially higher, as an estimated risk study from France shows [22], even though another French study did not find an increased risk of acquired HIV or hepatitis C infections following trans-vaginal US procedures [23]. Whatever the anticipated risk, known avoidable healthcare-acquired infections are likely to be reviewed with increasing scrutiny. In addition, all US practitioners have an ethical obligation to keep their patients as safe as possible, and hence should seek to minimise risk wherever possible.

Potential risks can be minimised by reducing transducer contamination, better US probe decontamination after every patient and by the use of appropriate US gel:

1. Reduction of US probe contamination (use of probe covers)
  Basic hygiene protocols such as hand washing, the use of gloves and keeping surfaces clean are essential first infection prevention and control measures. In addition, the use of transducer covers can lower contamination levels, particularly in interventional or endo-cavity US and whenever skin is not intact (i.e., when scanning close to wounds or stomas).
  The survey results show that a significant minority did not always use probe covers for endo-cavity scans (11 %) or interventional procedures (23 %), possibly due to a lack of awareness.
  The use of sterile probe covers is strongly recommended in the recently published *EFSUMB Guidelines on Interventional Ultrasound*, which also emphasise that subsequent transducer decontamination after every patient is needed [10]. The latter is important, as varying rates of probe cover perforations have been reported, ranging from 2 % [24] to 9 % of cases [25], and even up to 81 % in one older study [26]. Less frequent perforations were reported when condoms were used for trans-vaginal US rather than US probe covers [27, 28], but published studies are somewhat dated. Visual inspection may significantly underestimate contamination levels and is therefore unreliable [29]. Even when performing low-level decontamination, e.g., single-step wiping of probes with a disinfectant, persistent contamination was found on trans-vaginal US probes after probe covers had been used [8, 16].
2. Choice of US gel
  Gels used for medical purposes do not have bacteriostatic properties per se, and cannot be viewed as sterile unless specifically stated by the manufacturer. This was proven by studies in the 1990s [30, 31]. Contaminated US gel has been identified in routine cultures, and several reports of outbreaks of infection related to US gel have been published [18, 32–35].
  In this survey, a minority of practitioners used sterile gel sachets in direct patient contact for endo-cavity scans (30 %), whilst the majority used sterile gel for interventional procedures (77.5 %). Again, it appears that increasing awareness is important. It is also noted that a minority used sterile gel inside transducer covers. Considering the significant reported probe cover perforation rates (see above), it is debatable whether the use of sterile gel would lower infection rates.
  Recommendations regarding the safe use of US gels, including gel warmers, were published by Oleszkowicz et al. in 2012 [36]. The newly published *EFSUMB Guidelines on Interventional Ultrasound* recommend the use of sterile gel in interventional US, and state that new sachets should be opened for every patient [10]. We would agree with this advice.
3. US probe decontamination
  Complete decontamination of US transducers is challenging [8, 37]. A study testing the susceptibility of human papillomavirus, for example, showed that many disinfectants (including alcohol) may have limited to no effect [37], which is concerning and necessitates a general review of decontamination protocols used.
  Wiping probes with a dry towel resulted in persistent contamination [18]. Many potent and proven in vitro-effective chemical disinfectants, such as glutaraldehyde, can pose health hazards to staff in direct skin contact or through inhalation, as well as causing unhealthy work environments for staff and patients. The nature of the US probe surface must be considered as well, and its susceptibility to damage by chemicals—for example, alcohol [38]. Individual manufacturers should provide detailed information on which disinfectants are compatible, and should be prepared to provide further product testing upon request.
  Questionnaire responses reveal that not all practitioners disinfected the US transducers after every patient: 29 %, 11 % and 6 % of respondents did not disinfect the US probe after every patient when performing standard surface US, endo-cavity US and interventional procedures respectively. It seems that more needs to be done to emphasise the importance of adequate US transducer decontamination after every patient, and not just at the end of the US list.
  We acknowledge that implementing any additional infection prevention measures will not be cost-neutral. Capital investment for additional transducers and decontamination equipment may be needed, and there will be higher ongoing costs for probe covers and sterile gel. All staff working in US departments must be adequately trained in using the available resources as effectively as possible.
  Recommendations available to date include the European guidelines on interventional US mentioned above, which contain sections on infection prevention [10]. The British Medical Ultrasound Society (BMUS) published a survey and guidance in 2003 [9], but whilst very valuable at the time, this advice was never adopted UK-wide. NHS Health Scotland has just published a document, *Guidance for Decontamination of Semi-Critical Ultrasound Probes; Semi-invasive and Non-invasive Ultrasound Probes* [39], regarding recommended protocols. Several more recent publications from the USA and Canada relating to the safe use of medical gel and US probe decontamination are available, as well as guidance from Australasia and France [40–45]. Many local infection prevention and control guidelines also exist. However, to our knowledge, there are no specific published European or international guidelines available to advise general US practitioners on infection prevention and control matters. Up-to-date European expert recommendations, after a thorough literature review, would be helpful for protecting patients and ensuring that US examinations are as safe as possible.

## Limitations

The limitations of this survey include the relatively low response rate, with only 946 completed questionnaires (4.3 %). This may partly reflect the fact that not all radiologists performing US examinations (in 2015, just under half of the full ESR Radiologist Members chose US as one of their special interests), or in some departments, only the US lead radiologist may have completed the survey. There was unequal representation among European countries, with a larger number of responses from Italy, the UK, Spain and France. The vast majority of responses came from hospital settings, and a smaller number were received from private centres, which may not fully represent the second group. This survey focussed on infection prevention issues relating to US procedures. To keep the questionnaire as short as possible, the more wide-ranging basic infection prevention and control precautions such as hand washing, the use of gloves and protective drapes, and the cleaning of US machines (apart from transducers) and other surfaces were not explored. No question was included about the use of US gel warmers. Finally, we did not include options to allow for differences in the complexity of interventional procedures.

## Conclusion

These survey results underscore the importance of raising awareness amongst clinicians of the risks of infection associated with US, in particular when transducers are in contact with mucous membranes and potentially infected bodily fluids. Expert European recommendations are needed to educate clinicians, guide best practices and ensure that safe patient care is provided.

## Electronic supplementary material

Below is the link to the electronic supplementary material.

ESM 1 (PDF 99 kb)
