# Supplementary material for: Infection prevention and ultrasound probe decontamination practices in Europe: a survey of the European Society of Radiology
Source: Insights Imaging. 2016 Oct 24;7(6):841–7. doi: 10.1007/s13244-016-0528-z (PMC5110482; doi:10.1007/s13244-016-0528-z)
Supplement: Supplementary file 1 — (PDF 99 kb) [file 13244_2016_528_MOESM1_ESM.pdf]

Ultrasound (US) probe decontamination: Current practice

General Information

Thank you very much for having decided to take 5 minutes to complete our short survey!

* Country:

* City:

* Work place:

- ◯ Private centre
- ◯ Hospital
- ◯ Other (please specify):

* Size of the hospital:

- ◯ no beds
- ◯ less than 100 beds
- ◯ 100-499 beds
- ◯ 500-1,000 beds
- ◯ more than 1,000 beds

* Total number of US examinations per year:

- ◯ less than 3,000
- ◯ 3,000-10,000
- ◯ more than 10,000

* Department:

- ◯ Radiology
- ◯ Gynaecology/Obstetrics
- ◯ Urology
- ◯ Cardiology
- ◯ Other (please specify):

* Do you know of any cases of infection transmission through US procedures?

- ◯ no
- ◯ yes, please specify:

Ultrasound (US) probe decontamination: Current practice

Surface ultrasound on unbroken skin

Surface ultrasound on unbroken skin
For example standard trans-abdominal ultrasound

* Ultrasound gel used:

- ◯ Refill bottles
- ◯ Single use bottles
- ◯ Sterile gel sachets

* Ordinary probe decontamination:

- ◯ Wipe off US gel and probe disinfection only at end of US list (not after each patient)
- ◯ Wipe off US gel, disinfection after each patient with foam/wipe
- ◯ Probe cleaning in dedicated washer
- ◯ Other (please specify):

If you know the name of the used product, please specify it here:

* Infection control cases:
Which decontamination is performed?

- ◯ Wipe off US gel and disinfection with foam/wipe
- ◯ Probe cleaning in dedicated washer
- ◯ Other (please specify):

Ultrasound (US) probe decontamination: Current practice

Endo-cavity US probe decontamination

Endo-cavity US probe decontamination
For example trans-vaginal or trans-rectal US

Do you use a probe cover at all times?

- ◯ yes
- ◯ no

US gel used inside probe cover:

- ◯ Refill bottles
- ◯ Single use bottles
- ◯ Sterile gel sachets

US gel used in direct patient contact:

- ◯ Refill bottles
- ◯ Single use bottles
- ◯ Sterile gel sachets

Probe decontamination:

- ◯ Wipe off US gel and probe disinfection only at end of US list (not after each patient)
- ◯ Wipe off US gel, disinfection after each patient with foam/wipe
- ◯ 3-Step deep cleaning process (for example Tristel wipes)
- ◯ Probe cleaning in dedicated washer
- ◯ Other (please specify):

If you know the name of the used product, please specify it here:

Ultrasound (US) probe decontamination: Current practice

Probe decontamination after interventional procedures

Probe decontamination after interventional procedures
US probe in contact with patient body fluids like blood, for example US guided biopsies, intra-operative procedures

* Do you perform any interventional procedures?

- ◯ yes
- ◯ no

Ultrasound (US) probe decontamination: Current practice

Probe decontamination after interventional procedures

Probe decontamination after interventional procedures
US probe in contact with patient body fluids like blood, for example US guided biopsies, intra-operative procedures

* Do you use a probe cover at all times?

- ◯ yes
- ◯ no

* US gel used inside probe cover:

- ◯ Refill bottles
- ◯ Single use bottles
- ◯ Sterile gel sachets

* US gel used in direct patient contact:

- ◯ Refill bottles
- ◯ Single use bottles
- ◯ Sterile gel sachets

* Probe decontamination:

- ◯ Wipe off US gel and probe disinfection only at end of US list (not after each patient)
- ◯ Wipe off US gel, disinfection after each patient with foam/wipe
- ◯ 3-Step deep cleaning process (for example Tristel wipes)
- ◯ Probe cleaning in dedicated washer
- ◯ Other (please specify)

[ ]

If you know the name of the used product, please specify it here:

[ ]
